# Developmental Coordination Disorder, Motor Performance, and Daily Participation in Children with Attention Deficit and Hyperactivity Disorder

**DOI:** 10.3390/children8030187

**Published:** 2021-03-01

**Authors:** Rebeca Montes-Montes, Laura Delgado-Lobete, Sara Rodríguez-Seoane

**Affiliations:** 1 TALIONIS Research Group, Research Centre of the Galician University System, Centre for Information and Communications Technology Research (CITIC), Universidade da Coruña, 15008 A Coruña, Spain; rebeca.montes@udc.es; 2 Health Integration and Promotion Research Unit (INTEGRA SAÚDE), Faculty of Health Sciences, University of A Coruña, 15011 A Coruña, Spain; 3 Spanish Cerebral Palsy Association (ASPACE), 33394 Gijón, Spain; sarose79@gmail.com

**Keywords:** attention deficit hyperactivity disorder, developmental coordination disorder, comorbidity, motor performance, daily participation, activities of daily living, occupational therapy

## Abstract

Children with Attention Deficit and Hyperactivity Disorder (ADHD) often present with Developmental Coordination Disorder (DCD) or motor coordination problems that further impact their daily functioning. However, little is known about the prevalence of co-occurring DCD and ADHD in the Spanish context, and research about the impact of ADHD on performance and participation in motor-based activities of daily living (ADL) is scarce. The aims of this study were to explore the prevalence of co-occurring DCD in children with ADHD, and to examine differences in performance and participation in motor-based ADL between children with ADHD and typically developing children. We conducted a case-control study including 20 children with ADHD and 40 typically developing controls randomly matched for exact age and sex (males = 80%; mean age = 8, 9 (2, 3) years). Presence of probable DCD (p-DCD) was confirmed with the Developmental Coordination Disorder Questionnaire (DCDQ). The DCDDaily-Q was administered to assess performance and participation in ADL. A 75% prevalence of p-DCD was found in the ADHD group (OR = 27; *p* < 0.001). Children with ADHD showed poorer motor performance and less participation in ADL (*p* < 0.01; *d* = 0.9–1.4). These findings contribute to understand the functional consequences of ADHD in motor-based ADL and its relationship with DCD.

## 1. Introduction

Neurodevelopmental disorders are present in 4–18% of children, and these disorders significantly affect the child’s daily functioning [1,2,3,4]. Some of the most prevalent disorders reported in literature are Attention Deficit and Hyperactivity Disorder (ADHD), Developmental Coordination Disorder (DCD), Specific Learning Disorder, and Autism Spectrum Disorders [2,5,6,7,8,9,10,11]. ADHD affects approximately 4–9.5% school-aged children [2,5,6], while prevalence of DCD usually ranges between 5 and 6% [11]. However, recent research shows that up to 5–17% of children have risk of DCD or probable DCD [7,8,9,10].

ADHD is characterized by three core symptom domains (i.e., inattention, hyperactivity, and impulsivity) [11], but this disorder has further clinical and functional long-term impact on the child’s daily performance [12]. For instance, psychosocial and coordination problems are largely present in these children [5,12]. Moreover, it has been reported that up to 87% children with ADHD have at least one co-occurrent diagnosis [13], being DCD the most common co-occurrence [13]. According to several studies that have explored this association, the overlap between the two disorders ranges between 35% for general population and more than 50% in clinical samples [14,15,16,17]. Several conceptual frameworks have been proposed to explain the association and co-occurrence between different developmental disorders, such as the atypical brain development framework (ABD) [18]. This concept refers to the developmental variation of the brain, which may result in a variety of strengths and weaknesses in different skill areas and degrees [18]. According to this framework, atypical brain structure and function could affect different areas of behavior simultaneously (i.e., co-occurrent ADHD and DCD).

As previously mentioned, there are several neurodevelopmental disorders that affect the child’s performance and participation, and some of them usually present with ADHD. For instance, DCD is a motor neurodevelopmental condition with the highest co-occurrence with ADHD [13,14,15,16,17]. This disorder affects learning and execution of motor coordination skills, which significantly and persistently limits the child’s daily motor performance [7,11]. Available research has shown that children with DCD and children with poor motor coordination skills without DCD diagnosis (i.e., at risk of DCD) face restricted participation in activities of daily living (ADL) that are key to the child’s development and well-being, such as self-care activities, gross motor play activities, academic-related activities or social participation [19,20,21,22]. Thus, deficits in motor skills do not only have an impact on performance but on daily participation as well. 

Co-occurring deficits in motor performance of children with ADHD may have further consequences on their well-being and participation, as these children also encounter significant difficulties in performance and participation in those ADL that require attention or inhibitory control skills [12]. Previous studies indicate that 30–87% of children with ADHD or DCD will continue to face activity limitations and daily restricted participation during adulthood [23,24]. Moreover, children, adolescents, and adults with co-occurring ADHD and DCD have a much worse behavioral, psychosocial, and functional outcome than those individuals with ADHD or DCD alone [3,16,25].

As described by the Occupational Therapy Practice Framework and the International Classification of Functioning, Disability and Health for Children and Youth (ICF-CY), satisfactory participation in meaningful daily activities is a major component of health and well-being [26,27]. Previous studies have examined motor symptoms in ADHD, but research often focuses on motor competence (i.e., the ability to carry out a motor task) instead of motor performance (i.e., the actual performance of daily motor activities) [16,17]. According to the ICF-CY, performance of ADL describes what the child actually does in their usual environment [27], which in turn has an impact on their grade of participation or involvement in daily situations [19]. For instance, fine motor competence or ability can be assessed through a fine motor task in a standardized environment (i.e., threading lace or placing pegs according to the indications of an objective motor battery), while assessment of performance and participation focuses on actual environment and daily functioning (i.e., getting dressed or handling a key).

It is important to study the impact of ADHD on children’s motor daily functioning, and to identify potential limitations in motor performance and participation restrictions. In Spain, research interest in ADHD has increased during the last two decades, but studies regarding its association with DCD are scarce [17]. Because DCD is a highly unknown and underdiagnosed condition in Spain, there is not reliable information about the co-occurring prevalence of DCD in children with ADHD in the Spanish context, and Spanish children with ADHD are rarely screened and referred for co-occurring motor or coordination difficulties. Likewise, very little is known about their performance or participation in motor-based activities of daily living. While research suggests that approximately 60% of Spanish adolescents with ADHD have risk of poor motor competence [17], there is limited information available on this regard in Spanish school-age children. Differences in performance and participation in daily activities are influenced by cultural context even between western countries [9], and thus the impact of ADHD on performance and participation in motor-based activities in the Spanish context needs to be explored, especially considering that most self-care, school related or play activities during childhood do primarily involve motor coordination tasks [26]. 

Therefore, the aims of this study are: (a) to explore the prevalence of co-occurring DCD in Spanish children with a clinical diagnosis of ADHD in comparison to typically developing peers; and (b) to examine differences in performance and participation in motor-based activities of daily living between children with a clinical diagnosis of ADHD and typically developing children in the Spanish context.

## 2. Materials and Methods

### 2.1. Study Design, Participants and Procedure

We conducted a case-control study with a total sample of sixty children. This sample size was estimated in order to explore expected differences in prevalence of DCD between children with and without ADHD using a 1:2 case-control ratio with minimum bias (α < 0.05, power (1 − β) > 0.90) [7]. Children were eligible for the case group if they had a medical diagnosis of ADHD and did not have a co-occurrent diagnosed disorder or condition. Children were eligible for the control group if they did not have a parent-reported medical diagnosis of a neurodevelopmental or learning condition. 

The final sample was formed by twenty children with a medical diagnosis of ADHD (ADHD group; males = 80%; mean age = 8, 9 (2, 3) years), and forty children without any known developmental or learning disorders (TD group) randomly matched for exact age and sex. Detailed sociodemographic information of the sample is shown in Table A1 (Appendix A).

Children in the ADHD group were recruited from two private rehabilitation centers in Spain. Children in this group had a previous DSM-IV or DSM-5-based ADHD diagnosis given by a clinical practitioner (i.e., pediatrician, child neurologist, or psychologist), and they did not have co-occurring diagnosed disorders.

Children in the control group attended seven randomly selected elementary schools in three Spanish regions and were excluded beforehand if their parents reported a medically diagnosed neurodevelopmental or learning condition [11]. Children in this group were part of a larger, multicentric study evaluating risk of DCD in Spain [28]. Parents of participants were given the European Spanish versions of the Developmental Coordination Disorder Questionnaire (DCDQ) and the DCDDaily-Q via center or school intermediation. They anonymously completed both questionnaires at home and returned them to the center or school within a week.

Ethical approval for this study was granted by the Autonomic Research Ethics Committee of Galicia, Spain (code 2018-606).

### 2.2. Measures

#### 2.2.1. Assessment of Developmental Coordination Disorder

The European Spanish version of the DCDQ [29,30] was used to identify children at risk of DCD. The DCDQ is a well validated and widely used instrument to operationalize criterion B of the DSM-5 diagnostic criteria of DCD [7,11]. It comprises 15 items covering control during movement, fine motor/handwriting and general coordination during activities of daily living in children aged 5 to 15 years. Parents rate each item using a five-point Likert scale. Items scores are summed to provide total and subscale scores, where higher scores indicate better daily motor performance [31]. The DCDQ has demonstrated good cultural equivalence, internal consistency (Cronbach’s α = 0.907), construct validity and discriminant validity in Spanish children (sensitivity = 76.7%; specificity = 83.3%; Area Under the Curve = 0.872) [29,30].

Children who scored at or below age-specific 15th percentile in DCDQ total score (criterion B of the DSM-5 DCD diagnosis) were identified as having probable DCD (p-DCD) [30,31]. Criterion D (the motor skills deficits are not better explained by intellectual disability or by medical conditions affecting movement) was confirmed by only including children in the control group who had not a known diagnosis of learning, medical, or neurodevelopmental condition. Criterion A and criterion C were not specifically evaluated, and thus children were classified as having p-DCD instead of a formal diagnosis of DCD [32].

#### 2.2.2. Performance and Participation in Activities of Daily Living

The European Spanish version of the DCDDaily-Q [28,33] was used to assess performance and participation in motor-based daily activities. The DCDDaily-Q is a parent questionnaire that evaluates motor performance and level of participation in 23 activities of daily living (ADL) covering self-care, fine motor and gross motor play activities [34]. Parents rate motor performance for each activity using a three-point Likert scale. Children participation is rated using a four-point Likert scale in each activity. Items scores are summed to provide total (general ADL) and subscale scores (self-care, fine motor, and gross motor ADL) for motor performance and participation. Higher scores indicate poorer daily motor performance and participation. The DCDDaily-Q has been cross-culturally adapted to Spanish population and it shows good internal consistency (Cronbach’s α = 0.843), excellent construct validity, and good criterion validity with the DCDQ (*r* = 0.406–0.638) [28,33,34].

### 2.3. Data Analysis

Statistical analyses were performed using IBM SPSS version 25 (SPSS Inc., Chicago, IL, USA). A 1:2 case-control ratio was used to improve statistical power [35]. Differences in the prevalence of p-DCD between ADHD and control groups were assessed using Chi-square test. Odds ratio and their 95% confidence intervals were calculated to determine the magnitude of the association between ADHD and p-DCD. The data were examined for normality using skewness and kurtosis inspection [36]. Finally, differences in daily motor performance and participation between ADHD and control groups were explored with independent *t*-tests. In addition, the effect size of these differences was examined with Cohen’s *d* [37].

## 3. Results

### 3.1. Co-Occurrence of Probable Developmental Coordination Disorder

Prevalence of p-DCD was statistically higher in the ADHD group in comparison to control group (Table 1). Co-occurrence of ADHD and p- DCD was of 75%, while children without ADHD presented with 10% prevalence of p-DCD. Children with ADHD were twenty-seven times more likely to show p-DCD compared to children without ADHD (OR = 27, 95% CI = 6.4–114.7). In addition, children with ADHD reported lower scores in DCDQ subscales and total score (Table 2).

### 3.2. Differences in Daily Performance and Participation

Children with ADHD scored significantly higher in all subscales and total score of the DCDDaily-Q, indicating a poorer daily performance and less level of participation in self-care, fine motor, gross motor and general ADL than children without ADHD (Table 3). All differences were large (*d* > 0.8), and the strongest difference between groups was found for participation total score (*d* = 1.4, *p* < 0.001).

A descriptive analysis of the results of the DCDDaily-Q between ADHD children with and without p-DCD can be found in Table A2 (Appendix A).

## 4. Discussion

Co-occurrence between DCD and ADHD is highly common, but the impact of deficits in motor coordination skills on performance and participation in motor-based daily activities needs to be further explored, particularly in the Spanish context. Therefore, this study aimed to examine co-occurring DCD and differences in performance and participation between children with and without ADHD. 

### 4.1. Co-Occurrence of Probable Developmental Coordination Disorder and ADHD

In our sample, prevalence of p-DCD in the control group was 10%, which is consistent with previous research in epidemiological studies in Spain [8,9,10]. Co-occurrence of p- DCD in the ADHD group was of 75%, which was statistically higher in comparison to the control group. This finding is in line with previous research that has persistently reported that DCD is the most frequent co-occurring disorder with ADHD, and vice-versa [7,13]. While the most frequent reported overlap between both disorders in clinical samples is about 50% and higher [7], other studies have reported a co-occurrence as high as 74% in medically diagnosed ADHD samples [38].

A recent study involving Spanish high school students found an overlap between ADHD and risk of DCD of approximately 60% [17]. To the best of our knowledge, the present study is the first to explore co-occurrence of DCD in Spanish children with ADHD younger than thirteen years. This is important because prevalence and functional manifestations of DCD can vary with age [8]. Furthermore, Villa and colleagues assessed motor coordination ability to determine the risk of DCD in their sample, but they did not evaluate performance or participation in motor-based daily activities, which is a major diagnostic criterion of DCD [11]. While there are differences in methodology, sample characteristics and assessment of DCD between Villa and colleagues’ research and the present work, findings of both studies suggest that co-occurrence between ADHD and DCD in the Spanish context is similar to that reported in international literature in clinical samples [7,16,38]. As prevalence of DCD differs across countries, even in western countries [9], exploring the association between ADHD and DCD in the Spanish context contributes to the general understanding of the co-occurrence between these disorders.

Links and similarities between ADHD and DCD are well documented in scientific literature. There is substantial evidence that children with ADHD show poorer motor skills than their typically developing peers, affecting not only gross motor but also fine motor skills [39]. Children with attentional, impulsivity, or hyperactivity difficulties usually face problems in motor planning, coordination and execution as well [5]. Equally, children with DCD frequently display attention deficits and difficulties in inhibitory control, which leads to impulsivity and hyperactivity behavior with further consequences on performance and participation [7]. Furthermore, both disorders share common additional co-occurrences, such as learning and social difficulties, atypical sensory processing and internalizing mental health problems [3,5,7,40]. These similarities, alongside the high overlap between ADHD and DCD, have raised questions regarding a potential shared etiology of an atypical development of the brain with different manifestations, like it is proposed by the ABD framework (i.e., with children more likely to present attentional, impulsivity, and hyperactivity behavior and other children more likely to present motor coordination deficits) [18]. However, recent evidence from neuroimage research indicates that ADHD and DCD are most likely two different disorders with specific etiology, distinct neurophysiological mechanisms and separate brain underpinnings, even though their manifestation usually overlap [41,42]. Nevertheless, it is important to identify those children who may present with both ADHD and DCD, because they will be more likely to encounter greater daily difficulties and will need specific intervention approaches that address both the inattention, impulsivity and hyperactivity symptoms and the deficits in motor coordination skills.

### 4.2. Differences in Daily Performance and Participation

Apart from the high co-occurrence of ADHD and p-DCD, children in the ADHD group showed significant poorer performance and participation in motor-based self-care, fine motor, gross motor, and general ADL. This particular outcome contributes to the understanding of the impact of ADHD on motor-based performance and it makes a counterpart to the findings from previous research on motor competence in Spanish individuals with ADHD [17]. Overall, it can be concluded that children with attention, hyperactivity and impulsivity problems show lower levels of motor competence than their typically developing peers, especially in manual dexterity and balance [16,17], which in turn leads to limitations in motor performance on specific, complex daily living activities, and reduced participation. If confirmed, this outcome would suggest a further link to DCD, where deficits in motor skills lead to poorer execution and reduced participation in motor-based ADL [19,43]. 

These findings need to be interpreted cautiously given the limited sample size of this study. As most of the children in the ADHD group showed p-DCD as well, it cannot be concluded if the reported problems in motor performance and participation are better explained by the ADHD or the by the co-occurrent p-DCD. The descriptive analyses between children with ADHD and co-occurrent p-DCD and children with ADHD alone showed a persistent poorer performance and lower participation in the first group across all ADL, except for the gross motor play activities. If confirmed by future research, this finding could suggest that children with co-occurring ADHD and p-DCD have a higher risk of poor daily performance and participation due to motor coordination difficulties, which is in line with previous studies reporting worse outcomes in children with co-occurring ADHD and DCD [3,7,14]. Moreover, it would be needed to explore what is the role that the attentional and behavioral symptoms of children with co-occurring ADHD and DCD play in this process, and how this relationship evolves during adolescence and adulthood, given that long-term functional consequences of ADHD persist into adulthood even when the symptoms are treated [44].

### 4.3. Limitations and Implications for Research and Clinical Practice

The main limitation of this study is that we could only established presence of p-DCD and a formal diagnosis of DCD was not possible, as we only assessed two of the four DSM-5 diagnostic criteria of DCD [11,32]. However, we used a well-validated and recommended instrument to identify children at risk of DCD, which has been validated in the target population. Another limitation is that daily performance and participation was assessed through parent-reported measures, which can introduce bias. However, parental questionnaires are useful instruments to assess daily performance and participation, as parents can provide reliable information about children’s functioning in daily contexts that cannot be recorded in a clinical or standardized setting [45,46]. Finally, the small sample size used in this study is another limitation, as it does not allow the analysis of potential differences in motor performance and participation between children with ADHD and p-DCD and children with ADHD alone.

Findings from this study can be used in research and clinical practice. Future studies should explore the longitudinal display and outcomes of performance and participation in motor-based ADL in children with ADHD and co-occurring ADHD and DCD. In addition, future research can study the relationship between DCD, motor performance and participation, and the different ADHD domains (i.e., inattention, hyperactivity, and impulsivity). Regarding clinical implications, occupational therapists and other social and health practitioners can use our findings to systematically assess children with ADHD for risk of DCD or motor coordination problems, and to design tailored intervention approaches aimed to address the potential consequences of motor coordination deficits in daily performance and participation.

## 5. Conclusions

The present study provides relevant and novel information regarding the co-occurrence of ADHD and DCD in the Spanish context, as well as the impact of ADHD on performance and participation in motor-based daily living activities. These findings contribute to highlight the need to assess motor skills in children with ADHD. Given the complex nature of ADHD symptoms and its co-occurrence with other developmental disorders, these children should be referred to multidisciplinary teams that include occupational therapists, psychologists, and educational practitioners to address the inattention, impulsivity-hyperactivity, and coordination symptoms and functional outcomes of this disorder, moreover, if ADHD is present with DCD as well.

## Figures and Tables

**Table 1:** Prevalence of p-DCD in ADHD and control groups (*n* = 60).

	ADHD Group (*n* = 20) *N* (%)	Control Group (*n* = 40) *N* (%)	X^2^	*p*-Value	OR (95% CI)
p-DCD			26.03	<0.001	27 (6.4–114.7)
With p-DCD	15 (75.0)	4 (10.0)			
Without p-DCD	5 (25.0)	36 (90.0)			

ADHD = Attention Deficit Hyperactivity Disorder, p-DCD = probable Developmental Coordination Disorder, OR = odds ratio, CI = confidence interval.

**Table 2:** Differences in DCDQ subscales and total scores between ADHD and control groups (*n* = 60).

DCDQ	ADHD Group (*n* = 20) Mean (SD)	Control Group (*n* = 40) Mean (SD)	*p*-Value	*d*
Control during movement	22.6 (4.8)	28.4 (3.0)	<0.001	1.5
Fine motor/handwriting	12.9 (3.8)	18.3 (3.0)	<0.001	1.6
General coordination	16.4 (3.9)	23.2 (2.5)	<0.001	2.1
DCDQ total score	51.9 (9.4)	69.8 (7.1)	<0.001	2.2

DCDQ = Developmental Coordination Disorder Questionnaire, ADHD = Attention Deficit Hyperactivity Disorder, *d* = effect size.

**Table 3:** Differences in performance and participation between ADHD and control groups (*n* = 60).

	ADHD Group (*n* = 20) Mean (SD)	Control Group (*n* = 40) Mean (SD)	*p*-Value	*d*
**Motor Performance**				
Self-care ADL	16.1 (4.7)	12.1 (2.0)	<0.001	1.1
Fine motor ADL	13.2 (7.5)	8.4 (2.4)	0.001	0.9
Gross motor ADL	11.0 (2.8)	8.5 (2.2)	<0.001	1.0
DCDDaily-Q total score	40.2 (12.5)	29.0 (5.2)	<0.001	1.2
**Daily Participation**				
Self-care ADL	16.1 (3.7)	13.4 (2.2)	0.001	0.9
Fine motor ADL	11.8 (2.8)	9.1 (2.5)	<0.001	1.0
Gross motor ADL	14.5 (2.8)	11.5 (2.9)	<0.001	1.1
DCDDaily-Q total score	42.4 (6.7)	33.9 (5.7)	<0.001	1.4

ADHD = Attention Deficit Hyperactivity Disorder, ADL = activities of daily living, *d* = effect size.

## Data Availability

The data that support the findings of this study are available from the corresponding author, L.D.-L., upon reasonable request.

## Appendix A

**Table A1:** Sociodemographic variables of the sample (*n* = 60).

Sociodemographic Variables	ADHD Group (*n* = 20)	Control Group (*n* = 40)
Age (Mean (SD))	8.9 (2.3)	8.9 (2.3)
Males (*N* (%))	16 (80.0)	32 (80.0)
Females (*N* (%))	4 (20.0)	8 (20.0)
5 y.o. (*N* (%))	2 (10.0)	4 (10.0)
Males (*N* (%))	2 (100.0)	4 (100.0)
Females (*N* (%))	0 (0.0)	0 (0.0)
6 y.o. (*N* (%))	1 (5.0)	2 (5.0)
Males (*N* (%))	1 (100.0)	2 (100.0)
Females (*N* (%))	0 (0.0)	0 (0.0)
7 y.o. (*N* (%))	4 (20.0)	8 (20.0)
Males (*N* (%))	2 (50.0)	4 (50.0)
Females (*N* (%))	2 (50.0)	4 (50.0)
8 y.o. (*N* (%))	2 (10.0)	4 (10.0)
Males (*N* (%))	2 (100.0)	4 (100.0)
Females (*N* (%))	0 (0.0)	0 (0.0)
9 y.o. (*N* (%))	2 (10.0)	4 (10.0)
Males (*N* (%))	1 (50.0)	2 (50.0)
Females (*N* (%))	1 (50.0)	2 (50.0)
10 y.o. (*N* (%))	3 (15.0)	6 (15.0)
Males (*N* (%))	2 (66.7)	4 (66.7)
Females (*N* (%))	1 (33.3)	2 (33.3)
11 y.o. (*N* (%))	3 (15.0)	6 (15.0)
Males (*N* (%))	3 (100.0)	6 (100.0)
Females (*N* (%))	0 (0.0)	0 (0.0)
12 y.o. (*N* (%))	3 (15.0)	6 (15.0)
Males (*N* (%))	3 (100.0)	6 (100.0)
Females (*N* (%))	0 (0.0)	0 (0.0)

ADHD = Attention Deficit Hyperactivity Disorder, y.o. = years old.

**Table A2:** Descriptive analysis of daily motor performance and participation between ADHD children with and without p-DCD (*n* = 20).

	With p-DCD (*n* = 15) Mean (SD)	Without p-DCD (*n* = 5) Mean (SD)
**Motor Performance**		
Self-care ADL	16.9 (5.1)	13.6 (1.8)
Fine motor ADL	14.0 (8.6)	10.6 (1.5)
Gross motor ADL	10.9 (3.3)	11.2 (0.8)
DCDDaily-Q total score	41.8 (14.1)	35.4 (1.5)
**Daily Participation**		
Self-care ADL	16.3 (4.2)	15.4 (2.2)
Fine motor ADL	12.1 (3.1)	10.8 (1.6)
Gross motor ADL	14.1 (3.0)	15.8 (1.8)
DCDDaily-Q total score	42.5 (7.7)	42.0 (2.7)

p-DCD = probable Developmental Coordination Disorder, ADL = activities of daily living.
